# The interaction between estimated glomerular filtration rate and dietary magnesium intake and its effect on stroke prevalence: a cross-sectional study spanning 2003–2018

**DOI:** 10.3389/fnut.2024.1395685

**Published:** 2024-06-11

**Authors:** Chunhua Liu, Linan Qiu, Yuanyuan Zhang, Liping Chen, Huaqiang Wang, Huajian Lin, Yongjun Tao, Haiqin Ye

**Affiliations:** ^1^Lishui Hospital of Traditional Chinese Medicine Affiliated to Zhejiang University of Chinese Medicine, Lishui City, China; ^2^Department of Neurology, The First Affiliated Hospital of Wenzhou Medical University, Wenzhou, China; ^3^Lishui Central Hospital, Lishui City, China

**Keywords:** stroke, estimated glomerular filtration rate, chronic kidney disease, dietary magnesium, interaction

## Abstract

**Background:**

Despite the known associations of dietary magnesium intake and estimated glomerular filtration rate (eGFR) with cardiovascular diseases, their combined effects on stroke risk remain unclear. Therefore, this study aims to explore the associations of dietary magnesium intake and eGFR with stroke risk.

**Methods:**

The National Health and Nutrition Examination Survey (NHANES) data of 37,637 adult participants (≥18 years) from 2003 to 2018 was analyzed. Dietary magnesium intake was categorized as low (≤ 254 mg/day) and normal (> 254 mg/day) based on experimental data. Multiple logistic regression analyses and interaction tests were conducted to assess the associations of dietary magnesium intake and eGFR with stroke risk, with a focus on the interaction between different chronic kidney disease (CKD) stages based on eGFR levels and dietary magnesium intake. Additional analyses included multiplicative interaction analysis, restricted cubic spline analysis, and subgroup evaluations by age, sex, and ethnicity.

**Results:**

Dietary magnesium intake and eGFR were inversely correlated with the risk of stroke. Participants with low dietary magnesium intake had a higher stroke risk than those with normal magnesium intake (odds ratio [OR] 1.09, 95% confidence interval [CI]: 1.03–1.42). Likewise, low eGFR was associated with an elevated stroke risk compared with normal eGFR (OR 1.56, 95% CI: 1.15–2.13). Furthermore, the two factors showed a multiplicative interaction effect on stroke risk (OR 1.05, 95% CI: 1.01–1.09). We observed a significant interaction between stage G3 CKD and low dietary magnesium intake (OR 1.05, 95% CI: 1.01–1.09), suggesting a potential association with stroke risk. However, similar associations were not observed for stages G4 and G5, possibly due to the smaller number of participants with G4 and G5 CKD. The restricted cubic spline analysis revealed a non-linear relationship between dietary magnesium intake, eGFR, and stroke risk. The interaction between magnesium deficiency and low eGFR persisted in participants aged >60 years, as well as in females, non-Hispanic Black people, and people of other races.

**Conclusion:**

Dietary magnesium intake and eGFR correlate negatively with stroke prevalence. Moreover, there was an interaction between dietary magnesium intake and stroke prevalence across different CKD stages. Further large-scale prospective studies are needed to analyze the potential relationship between dietary magnesium intake, eGFR, and stroke.

## 1 Introduction

Stroke is one of the leading causes of death and disability worldwide, and the fourth leading cause in the United States (1, 2). Stroke incidence increased by 70%, and its global prevalence surged by 85% from 1990 to 2019 (1). Every year, about 795,000 Americans suffer a stroke, resulting in approximately 185,000 deaths (3, 4). Most survivors of stroke face physical or psychological sequelae, which seriously affect their quality of life.

Previous studies have indicated that traditional stroke risk factors include hypertension, diabetes mellitus, smoking, and high cholesterol. Despite their recognized impact, these risk factors cannot fully account for all cases of stroke, pointing to inherent limitations in our current understanding (5, 6). For instance, some studies have found that a certain proportion of patients with stroke do not exhibit these traditional risk factors (7, 8). Therefore, identifying additional clinically significant risk factors is crucial for improving primary stroke prevention and alleviating the associated disease burden.

Magnesium functions as a cofactor for multiple enzymes involved in metabolic pathways, such as glycolysis, signal transduction, ion channel regulation, and membrane potential. Furthermore, as a natural calcium antagonist, magnesium regulates blood flow, vasoconstriction, and blood pressure (9, 10). Studies have shown that magnesium deficiency may increase the risk of hypertension, diabetes mellitus, metabolic syndrome, coronary heart disease, and even mortality (11–13). A meta-analysis of prospective cohort studies demonstrated a 6% reduction in all-cause mortality and a 5% reduction in cancer mortality for every 100 mg/day increase in dietary magnesium intake (14). In addition, a 15-year follow-up study found that a magnesium-rich diet lowered the risk of stroke (15). A recent study has explored the relationship between dietary magnesium intake and the risk of stroke using the National Health and Nutrition Examination Survey (NHANES) dataset (16). However, this study focused solely on the association between dietary magnesium intake and stroke risk without considering other potential factors, such as estimated glomerular filtration rate (eGFR).

Chronic kidney disease (CKD) is a global health concern due to its rising incidence, poor prognosis, and high treatment costs (17). Declining kidney function may increase stroke risk by altering the cerebral vasculature and reducing cerebral blood flow, which significantly impacts brain function (18). The eGFR is a key indicator of renal function, and the normal eGFR is above 90 mL/min/1.73 m^2^. Although *post hoc* analysis did not reveal any association between eGFR and stroke risk after adjusting for confounding factors (19), a meta-analysis showed that baseline eGFR below 60 mL/min/1.73 m^2^ (corresponding to CKD stages 3 to 5) is an independent risk factor of stroke (20). In addition, results from a prospective cohort study involving more than 400,000 participants from UK Biobank showed that the risk of total stroke and ischemic stroke increased exponentially when the eGFR was less than 75 mL/min/1.73 m^2^ (21). Overall, these findings suggest a possible link between eGFR and cerebrovascular changes.

Recent studies have shown that enhancing magnesium intake can reverse vascular calcification and enhance kidney function in rats (22, 23). Moreover, in rat models, magnesium supplementation, either alone or in conjunction with n-acetylcysteine, may reduce ischemia/reperfusion injury (24). Previous research has indicated a connection between magnesium deficiency and a heightened risk of CKD (25). Moreover, magnesium has been shown to protect against renal injury resulting from hyperphosphatemia (26). Therefore, dietary magnesium deficiency in conjunction with low eGFR may elevate stroke risk. Consequently, the combination of increased magnesium intake and effective CKD management may reduce this risk. Previous studies have not investigated the potential interaction between dietary magnesium intake and eGFR in relation to stroke risk. Therefore, this study aims to extend the existing literature by exploring the combined effects of dietary magnesium intake and eGFR on stroke risk in a large population. By incorporating eGFR as a covariate and examining potential interactions, our goal is to deepen the understanding of stroke risk factors. Previous studies have investigated the association between dietary magnesium intake and stroke risk (16). However, these studies were limited to the period from 2007 to 2018. In contrast, our study covers a broader timeframe from 2003 to 2018. By extending the study period and increasing the sample size, we further explored the relationship between dietary magnesium intake, eGFR, and stroke risk, deepening our understanding of the long-term trends and effects of dietary magnesium intake and eGFR on stroke. At the same time, we analyzed the interaction between different CKD stages and dietary magnesium intake in stroke risk. The results indicate an interaction between dietary magnesium intake and eGFR in stroke risk, indicating that maintaining normal-range dietary magnesium intake could be especially beneficial for patients with G3 CKD. However, whether normal dietary magnesium intake poses risks in the CKD population due to decreased renal excretion capabilities requires further investigation.

## 2 Methods

### 2.1 Data sources

The NHANES is designed to assess the health and nutritional status of the US population to investigate disease prevalence and identify risk factors. The NHANES was approved by the National Center for Health Statistics Institutional Review Board, and all participants provided written informed consent before the survey. For this study, NHANES data spanning eight consecutive periods from 2003 to 2018, including a total of 80,312 participants, were retrieved. The cross-sectional analysis excluded participants aged <18 years (*n* = 32,549), as well as those with missing eGFR and stroke questionnaire data (*n* = 4,595), missing magnesium intake data (*n* = 2,558), and incomplete covariate data (*n* = 2,973). Finally, the data of 37,637 participants were included in the study.

### 2.2 Determination of dietary magnesium intake

The dietary information of the study participants was obtained through 24-h dietary recalls by the mobile examination center through in-person and telephone interviews conducted 3–10 days later. The participants were asked about the types and amounts of food, drink, and water consumed in the 24 h preceding the interview. Dietary magnesium intake was averaged over the two recall periods; the value of the first recall was used if the second recall was unavailable. We used the estimated average dietary magnesium intake among our study population as the classification threshold. Despite a slight deviation from WHO’s recommended intake levels, we chose this threshold considering the overall intake of dietary magnesium among participants. This allowed us to categorize daily dietary magnesium intake into normal (>254 mg/day) and low (≤254 mg/day) groups (27).

### 2.3 Measurement of eGFR

In this study, we did not measure urine albumin-to-creatinine ratio (UACR) at baseline due to data gaps. Hence, CKD diagnosis relied solely on eGFR values. The diagnosis of CKD requires either albuminuria or an eGFR lower than 60 mL/min/1.73 m^2^ (28). The Chronic Kidney Disease Epidemiology Collaboration (CKD-EPI) equation was used to calculate the standardized creatinine-based eGFR for all participants. This study categorized participants into CKD and non-CKD groups based on their eGFR values. CKD was defined as an eGFR below 60 mL/min/1.73 m^2^ (29). Additionally, we staged the patients with CKD based on the eGFR into G3a (45–59 mL/min/1.73 m^2^), G3b (30–44 mL/min/1.73 m^2^), G4 (15–29 mL/min/1.73 m^2^), and G5 (<15 mL/min/1.73 m^2^). The CKD-EPI equation is as follows: eGFR = 141 × min (SCr/K, 1)^a^ × max (SCr/K, 1)^−1.209^ × 0.993^age^ × 1.018 [in females]. SCr indicates serum creatinine, K is 0.7 for females and 0.9 for males, a is −0.329 for females and − 0.411 for males, “min” indicates the minimum of SCr/K or 1, and “max” indicates the maximum of SCr/K or 1 (29).

### 2.4 Definition of stroke

We utilized a medical conditions questionnaire to identify instances of stroke. Participants were considered to have had a stroke if they answered affirmatively to the question: “Has a doctor or other healthcare professional ever diagnosed you with a stroke?” The validity of self-reported stroke has been established in prior studies, and in our research, stroke was treated as an outcome variable (30, 31).

### 2.5 Covariates

The covariates included age, sex, race/ethnicity, poverty-to-income ratio (PIR), body mass index (BMI), level of education, smoking status, alcohol consumption, diabetes mellitus, hypertension, cardiovascular disease (CVD), triglyceride (TG) concentration, total cholesterol (TC) concentration, total energy intake, and dietary fiber intake. Participants were categorized by racial groups, including Mexican Americans, non-Hispanic Black people, non-Hispanic Whites, other Hispanics, and people of other races. Income was assessed using the family PIR, which juxtaposes family income against size (32), serving as an indicator for evaluating income level and eligibility for federal nutrition assistance programs. For example, a PIR of 130% indicates potential eligibility for programs like the Supplemental Nutrition Assistance Program. Previous studies have used a PIR of 350% to differentiate middle-income from high-income families (33, 34). Therefore, this analysis utilized three PIR categories: < 1.30, 1.30–3.5, and > 3.5. In a total sample of 37,637 individuals, there were 2,979 missing PIR data entries, with 2,871 from the non-stroke group (approximately 8%) and 108 from the stroke group (approximately 19%). Based on the BMI, the subjects were categorized as underweight (BMI < 18.5 kg/m^2^), normal (BMI 18.5 to <25 kg/m^2^), overweight (BMI 25 to <30 kg/m^2^), and obese (BMI ≥ 30 kg/m^2^). A total of 408 individuals had missing BMI data, including 335 from the non-stroke group (<0.1%) and 73 from the stroke group (<5%). The level of education was classified as high school or below or above high school. A total of 36 participants had missing data for the level of education, including 33 from the non-stroke group (<0.1%) and 3 from the stroke group (<0.1%). Furthermore, the participants were categorized based on their smoking status into three groups: current (smoked ≥100 cigarettes in life and currently smoking), former (smoked ≥100 cigarettes in life and currently no longer smoking), and non-smokers (smoked or smoked <100 cigarettes in life). The alcohol intake of the participants was determined through a questionnaire, and the participants were accordingly classified into the non-drinker, 1–5 drinks/month, 5–10 drinks/month, and 10+ drinks/month groups. Overall, 6,298 individuals had missing data for alcohol consumption, including 6,024 from the non-stroke group (16%) and 280 from the stroke group (19%). Diabetes mellitus was diagnosed as a glycated hemoglobin of >6.5% or a fasting blood glucose concentration of ≥7 mmol/L, and hypertension was self-reported in the questionnaire. The total number of missing data entries for hypertension was 91, including 88 from the non-stroke group (<0.1%) and 3 from the stroke group (<0.1%). In this study, cardiovascular disease (CVD) includes coronary heart disease, heart failure, and angina. The total number of missing CVD data entries is 59, with 50 (<0.1%) in the non-stroke group and 9 (<0.1%) in the stroke group.

### 2.6 Statistical analysis

Given the complex sampling design of the NHANES database, a weighted analysis was conducted using interview weights (WTMEC2YR) and sampling weights for study design variables (SDMVPSU and SDMVSTRA). Continuous variables were expressed as mean ± standard deviation (SD), and compared between groups using Student’s t-test. Categorical data were presented as numbers and ratios [*n* (%)], and compared using the Rao-Scott chi-square test. Statistical analysis was conducted using SPSS (version 23.0) and R (version 4.1.3) software.

The correlation between magnesium intake, eGFR, and stroke risk was evaluated by applying weighted logistic regression to adjust for confounding factors. Three models were constructed as follows: Model 1 adjusted for age and gender; Model 2 further incorporated race, education level, and PIR based on Model 1; and Model 3 adjusted for variables that were significant in the univariate logistic regression analysis, including age, gender, race, level of education, PIR, BMI, smoking, drinking, hypertension, diabetes mellitus, TC, TG, total energy intake, dietary fiber intake, and CVD. The odds ratios (OR) and 95% confidence intervals (CI) were calculated. We utilized weighted multiple regression analysis to assess the interaction between daily magnesium intake <254 mg and low eGFR <60 mL/min/1.73 m^2^ on stroke risk, examining their multiplicative interaction through OR testing. Subsequently, we analyzed the interaction between different CKD stages and dietary magnesium intake of <254 mg in the context of stroke risk. The interaction was considered significant when the 95% CI of the product excluded 1.

Furthermore, a restricted cubic spline model was used to examine the dose–response relationship between dietary magnesium intake and stroke risk, as well as the extent–response relationship between eGFR levels and stroke risk. The three nodes of the model were situated at the 5th, 50th, and 95th percentiles of dietary magnesium intake. Finally, subgroup analyses were conducted based on age, gender, and race to evaluate the combined effects of inadequate magnesium intake and low eGFR on stroke risk in different populations. We utilized multiple imputation methods to address missing values in this study. Statistical significance was set at *p* < 0.05.

## 3 Results

### 3.1 Baseline characteristics

**Figure 1:**
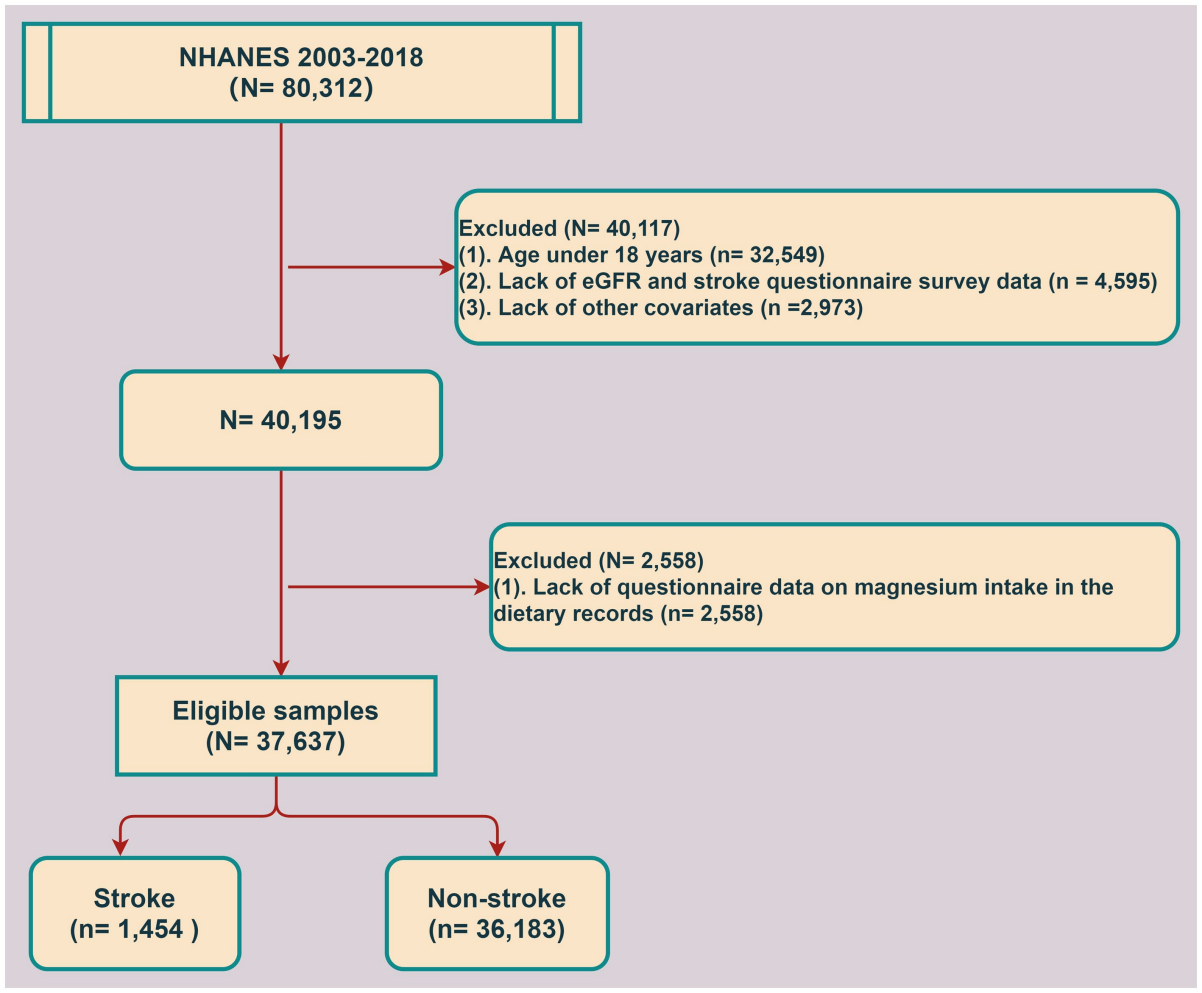
Participant screening process.

We analyzed the NHANES data of 37,637 participants after excluding those with missing data (Figure 1). The baseline characteristics of the stroke and non-stroke groups are summarized in Table 1. There were significant differences between the groups in terms of age, sex, level of education, PIR, BMI, smoking, alcohol consumption, hypertension, diabetes mellitus, CVD, TC, TG, eGFR, total calorie intake, fiber intake, dietary magnesium intake, and CKD staging based on eGFR. The stroke group had a higher proportion of individuals with low eGFR compared to the non-stroke group. Likewise, the proportion of subjects with daily magnesium intake >254 mg was also lower in the stroke group. In addition, the stroke group had higher TC, TG, and BMI compared to the non-stroke group. Conversely, both total calorie intake and fiber intake were lower in the stroke group than in the non-stroke group. Overall, low eGFR and inadequate magnesium intake were more prevalent in the stroke group (Table 1).

**Table 1:** Characteristics of participants.

Characteristic	N^1^	Overall N = 37,637 (100%)^2^	Group		*P* Value^3^
			Non-stroke N = 36,183 (97%)^2^	Stroke N = 1,454 (2.8%)^2^	
Age (years), Median (Mean, SD)	37,637	46 (47, 17)	46 (47, 17)	67 (65, 14)	<0.001
Age group, n (%)	37,637				<0.001
18–39 years		12,767 (34%)	12,706 (35%)	61 (4%)	
40–59 years		12,148 (32%)	11,836 (33%)	312 (22%)	
>= 60 years		12,722 (34%)	11,641 (32%)	1,081 (74%)	
Gender, n (%)	37,637				0.005
Female		19,329 (51%)	18,596 (51%)	733 (50%)	
Male		18,308 (49%)	17,587 (49%)	721 (50%)	
Race, n (%)	37,637				<0.001
Mexican American		6,187 (16%)	6,041 (17%)	146 (10%)	
Other Hispanic		3,325 (9%)	3,246 (9%)	79 (5%)	
Non-Hispanic White		16,772 (45%)	16,014 (44%)	758 (52%)	
Non-Hispanic Black people		7,724 (21%)	7,351 (20%)	373 (26%)	
Other Races		3,629 (9%)	3,531 (10%)	98 (7%)	
Education level, n (%)	37,637				<0.001
<=High school		9,408 (25%)	8,899 (25%)	509 (35%)	
>High school		28,193 (75%)	27,251 (75%)	942 (65%)	
Missing data		36 (<0.1%)	33 (<0.1%)	3 (<0.1%)	
Ratio of family income to poverty, Median (Mean, SD)	34,658	3.03 (3.02, 1.63)	3.08 (3.04, 1.63)	1.97 (2.34, 1.46)	<0.001
Ratio of family income to poverty, n (%)	34,658				<0.001
< 1.30		10,958 (29%)	10,404 (29%)	554 (38%)	
1.30–3.5		12,997 (35%)	12,439 (34%)	558 (38%)	
> 3.5		10,703 (28%)	10,469 (29%)	234 (16%)	
Miss		2,979 (8%)	2,871 (8%)	108 (19%)	
BMI, Median (Mean, SD)	37,229	28 (29, 7)	28 (29, 7)	29 (30, 7)	<0.001
BMI group, n (%)	37,229				<0.001
Underweight (<18.5)		564 (1%)	546 (1%)	18 (1%)	
Normal (18.5 to <25)		10,111 (27%)	9,794 (27%)	317 (22%)	
Overweight (25 to <30)		12,460 (33%)	12,010 (33%)	450 (32%)	
Obese (30 or greater)		14,094 (37%)	13,498 (37%)	596 (41%)	
Missing data		408 (<2%)	335 (<0.1%)	73 (<5%)	
Waist circumference, Median (Mean, SD)	36,419	98 (99, 16)	98 (99, 16)	103 (104, 16)	<0.001
TG (mg/dL), Median (Mean, SD)	18,079	104 (129, 107)	104 (129, 107)	114 (140, 101)	<0.001
TC (mg/dL), Median (Mean, SD)	37,626	192 (195, 42)	192 (195, 42)	181 (187, 46)	<0.001
Smoking status, n (%)	37,637				<0.001
Current		7,760 (21%)	7,421 (20%)	339 (24%)	
Former		9,325 (25%)	8,782 (25%)	543 (37%)	
Non-smoker		20,552 (54%)	19,980 (55%)	572 (39%)	
Alcohol consumption status, n (%)	31,339				<0.001
Non-drinker		9,026 (24%)	8,603 (24%)	423 (29%)	
1–5 drinks/month		15,497 (41%)	14,901 (41%)	596 (41%)	
5–10 drinks/month		2,422 (6%)	2,381 (7%)	41 (3%)	
10+ drinks/month		4,388 (12%)	4,274 (12%)	114 (8%)	
Missing data		6,298 (17%)	6,024 (16%)	280 (19%)	
Diabetes, n (%)	37,637				<0.001
Yes		6,301 (17%)	5,742 (16%)	559 (38%)	
No		31,336 (83%)	30,441 (84%)	895 (62%)	
Hypertension group, n (%)	37,546				<0.001
Yes		13,418 (36%)	12,311 (34%)	1,107 (76%)	
No		24,128 (64%)	23,784 (66%)	344 (24%)	
Missing data		91 (<0.1%)	88 (<0.1%)	3 (<0.1%)	
Cardiovascular disease, n (%)	37,578				<0.001
Yes		1,662 (4%)	1,360 (4%)	302 (21%)	
No		35,916 (96%)	34,773 (96%)	1,143 (79%)	
Missing data		59 (<0.1%)	50 (<0.1%)	9 (<0.1%)	
Dietary magnesium intake, Median (Mean, SD)	37,637	293 (322, 164)	294 (324, 164)	252 (283, 163)	<0.001
Dietary total energy intake, Median (Mean, SD)	37,637	1,988 (2,122, 862)	2,000 (2,132, 863)	1,676 (1,777, 754)	<0.001
Dietary fiber intake, Median (Mean, SD)	37,637	15 (17, 9)	15 (17, 9)	13 (14,7)	<0.001
Magnesium intake group, n (%)	37,637				<0.001
Mg ≤ 254 mg/day		17,003 (45%)	16,138 (45%)	865 (59%)	
Mg > 254 mg/day		20,634 (55%)	20,045 (55%)	589 (41%)	
eGFR^4^ (mL/min/1.73m^2^), Median (Mean, SD)	37,637	96 (94, 22)	96 (95, 22)	72 (73, 25)	<0.001
eGFR group, n (%)	37,637				<0.001
eGFR ≥ 60 mL/min/1.73m^2^		34,208 (91%)	33,247 (92%)	961 (66%)	
eGFR < 60 mL/min/1.73m^2^		3,429 (9%)	2,936 (8%)	493 (34%)	
45 < eGFR < 59 mL/min/1.73m^2^ (G^5^3a)		2,186 (5.8%)	1,924 (5.4%)	262 (18%)	
30 < eGFR < 44 mL/min/1.73m^2^ (G^5^3b)		859 (2.2%)	707 (1.9%)	152 (10.6%)	
15 < eGFR < 29 mL/min/1.73m^2^ (G^5^4)		270 (0.7%)	213 (0.5%)	57 (3.9%)	
eGFR < 15 mL/min/1.73m^2^ (G^5^5)		114 (0.3%)	92 (0.2%)	22 (1.5%)	

1 N not Missing (unweighted).

2 Median (IQR) for continuous; n (%) for categorical.

3 Wilcoxon rank-sum test for complex survey samples; chi-squared test with Rao & Scott's second-order correction.

4 Estimated glomerular filtration rate.

5 Chronic kidney disease Stage.

### 3.2 Dietary magnesium intake and eGFR are correlated with stroke risk

The association of dietary magnesium intake and eGFR with stroke risk is summarized in Table 2. Multivariable logistic regression showed that low dietary magnesium intake (≤ 254 mg/day) was associated with a significant increase in stroke risk (Model 1: OR = 1.66, 95% CI: 1.46–1.89, *p* < 0.001; Model 2: OR = 1.37, 95% CI: 1.19–1.58, p < 0.001; Model 3: OR = 1.09, 95% CI: 1.03–1.42, *p* = 0.043). Furthermore, participants with eGFR<60 mL/min/1.73 m^2^ showed a positive association with stroke risk compared to those with eGFR ≥60 mL/min/1.73 m^2^ (Model 1: OR = 2.11, 95% CI: 1.78–2.49, p < 0.001; Model 2: OR = 1.90, 95% CI: 1.59–2.26, p < 0.001; Model 3: OR = 1.56, 95% CI: 1.15–2.13, *p* = 0.005).

**Table 2:** Association between eGFR and dietary magnesium intake in the context of stroke risk.

Variable	Model 1 OR (95% CI)	p-value	Model 2 OR (95% CI)	p-value	Model 3 OR (95% CI)	p-value
eGFR ≥ 60 mL/min/1.73m^2^						
eGFR < 60 mL/min/1.73m^2^	2.11 (1.78–2.49)	<0.001	1.90 (1.59–2.26)	<0.001	1.56 (1.15–2.13)	0.005
Mg > 254 mg/day						
Mg ≤ 254 mg/day	1.66 (1.46–1.89)	<0.001	1.37 (1.19–1.58)	<0.001	1.09 (1.03–1.42)	0.043

OR = Odds Ratio, CI = Confidence Interval, eGFR = estimated glomerular filtration rate; Model 1, adjusted for age and gender; Model 2, adjusted for age, gender, race, education, and the ratio of family income to poverty; Model 3, adjusted for age, gender, race, education, the ratio of family income to poverty, BMI, alcohol consumption status, smoking status, hypertension, diabetes, triglyceride level, total cholesterol level, energy intake, dietary fiber intake, and cardiovascular disease.

### 3.3 Dietary magnesium deficiency and low eGFR synergistically increase stroke risk

As shown in Table 3, there was a significant multiplicative interaction between low dietary magnesium intake (≤ 254 mg/day) and eGFR <60 mL/min/1.73 m^2^ in the context of stroke risk (OR = 1.05, 95% CI: 1.01–1.09), implying a potential synergistic effect. Furthermore, the likelihood ratio test statistic was 1.456 (*p* = 0.018), indicating an interaction between these two factors in stroke risk. Following that, we evaluated the interaction between various CKD stages and dietary magnesium intake in the context of stroke risk. Specifically, we noted a significant interaction between low dietary magnesium intake and stroke risk in stage G3 CKD (OR = 1.05, 95% CI: 1.01–1.09), but such an interaction was not observed in patients with stage G4 or G5 CKD (Table 4).

**Table 3:** Interaction effects of dietary magnesium intake and eGFR in the context of stroke risk.

Variable	Magnesium > 254 (mg/day)		Magnesium ≤ 254 (mg/day)		Interaction (eGFR < 60 mL/min/1.73m^2^* Mg ≤ 254 mg/day)	
	OR (95% CI)	p- Value	OR (95% CI)	p- Value	OR (95% CI)	p for interaction
Subgroups					1.05 (1.01–1.09)	0.018
eGFR ≥ 60 mL/min/1.73m^2^						
eGFR < 60 mL/min/1.73m^2^	1.04 (1.00–1.07)	0.032	1.07 (1.04–1.11)	<0.001		

OR = Odds Ratio, CI = Confidence Interval, eGFR = estimated glomerular filtration rate; Model adjusted for age, gender, race, education, the ratio of family income to poverty, BMI, alcohol consumption status, smoking status, hypertension, diabetes, triglyceride level, total cholesterol level, energy intake, dietary fiber intake, and cardiovascular disease.

**Table 4:** Interaction effects of dietary magnesium intake and different chronic kidney disease stages in the context of stroke risk.

Variable	Magnesium > 254 (mg/day)		Magnesium ≤ 254 (mg/day)		Interaction (Different Chronic kidney disease Stages* Mg ≤ 254 mg/day)	
	OR (95% CI)	p- Value	OR (95% CI)	p- Value	OR (95% CI)	p for interaction
Subgroups					1.05 (1.01–1.09)	0.025
eGFR ≥ 60 mL/min/1.73m^2^						
30 ≤ eGFR < 59 mL/min/1.73m^2^ (G3)	1.03 (1.01–1.07)	0.06	1.06 (1.03–1.11)	<0.001		
15 ≤ eGFR < 30 mL/min/1.73m^2^ (G4)	1.14 (0.92–1.37)	0.21	1.15 (1.04–1.28)	0.007	1.04 (0.84–1.29)	0.7
eGFR < 15 mL/min/1.73m^2^ (G5)	1.43 (0.93–2.16)	0.11	1.05 (0.91–1.20)	0.5	0.74 (0.51–1.06)	0.10

OR = Odds Ratio, CI = Confidence Interval, eGFR = estimated glomerular filtration rate, G = Chronic kidney disease Stage; Model adjusted for age, gender, race, education, the ratio of family income to poverty, BMI, alcohol consumption status, smoking status, hypertension, diabetes, triglyceride level, total cholesterol level, energy intake, dietary fiber intake, and cardiovascular disease.

We then conducted subgroup analyses based on age, sex, and race, and the results are summarized in Table 5. A significant interaction between low dietary magnesium intake and eGFR <60 mL/min/1.73 m^2^ in stroke risk was evident in participants aged >60 years, and females. Furthermore, a significant multiplicative interaction between both factors was observed among non-Hispanic Black people and other races.

**Table 5:** Interaction effect between dietary magnesium intake and eGFR in the context of stroke risk in different demographic subgroups.

Subgroup		Model 1	p-value	Model 2	p-value	Model 3	p-value
		OR (95% CI)		OR (95% CI)		OR (95% CI)	
Age Subgroup	18 - 39 years						
	eGFR < 60 mL/min/1.73m^2^ * Mg ≤ 254 mg/day	1.14 (0.98–1.33)	0.083	1.15 (0.98–1.35)	0.084	1.17 (0.95–1.43)	0.14
	40 - 59 years						
	eGFR < 60 mL/min/1.73m^2^ * Mg ≤ 254 mg/day	1.06 (0.99–1.13)	0.1	1.06 (0.99–1.13)	0.11	1.05 (0.94–1.17)	0.4
	>= 60 years						
	eGFR < 60 mL/min/1.73m^2^ * Mg ≤ 254 mg/day	1.05 (1.02–1.08)	0.004	1.05 (1.01–1.08)	0.005	1.04 (1.00–1.08)	0.046
Gender Subgroup	Female						
	eGFR < 60 mL/min/1.73m^2^ * Mg ≤ 254 mg/day	1.06 (1.02–1.10)	0.003	1.06 (1.02–1.10)	0.004	1.05 (1.00–1.11)	0.049
	Male						
	eGFR < 60 mL/min/1.73m^2^ * Mg ≤ 254 mg/day	1.05 (1.02–1.09)	0.003	1.05 (1.02–1.09)	0.004	1.04 (0.99–1.10)	0.13
Race Subgroup	Mexican American						
	eGFR < 60 mL/min/1.73m^2^ * Mg ≤ 254 mg/day	1.01 (0.92–1.10)	0.8	1.02 (0.93–1.12)	0.7	0.93 (0.78–1.11)	0.4
	Other Hispanic						
	eGFR < 60 mL/min/1.73m^2^* Mg ≤ 254 mg/day	1.00 (0.92–1.08)	0.9	0.98 (0.90–1.07)	0.6	1.01 (0.92–1.10)	0.8
	Non-Hispanic White						
	eGFR < 60 mL/min/1.73m^2^ * Mg ≤ 254 mg/day	1.05 (1.02–1.08)	0.001	1.05 (1.02–1.08)	0.002	1.04 (1.00–1.08)	0.08
	Non-Hispanic Black						
	eGFR < 60 mL/min/1.73m^2^ * Mg ≤ 254 mg/day	1.04 (0.98–1.11)	0.2	1.05 (0.98–1.12)	0.15	1.09 (1.01–1.18)	0.042
	Other						
	eGFR < 60 mL/min/1.73m^2^ * Mg ≤ 254 mg/day	1.19 (1.05–1.34)	0.007	1.18 (1.04–1.33)	0.011	1.24 (1.01–1.53)	0.04

OR = Odds Ratio, CI = Confidence Interval, eGFR = estimated glomerular filtration rate; Model 1, adjusted for age and gender; Model 2, adjusted for age, gender, race, education, and the ratio of family income to poverty; Model 3, adjusted for age, gender, race, education, the ratio of family income to poverty, BMI, alcohol consumption status, smoking status, hypertension, diabetes, triglyceride level, total cholesterol level, energy intake, dietary fiber intake, and cardiovascular disease.

**Figure 2:**
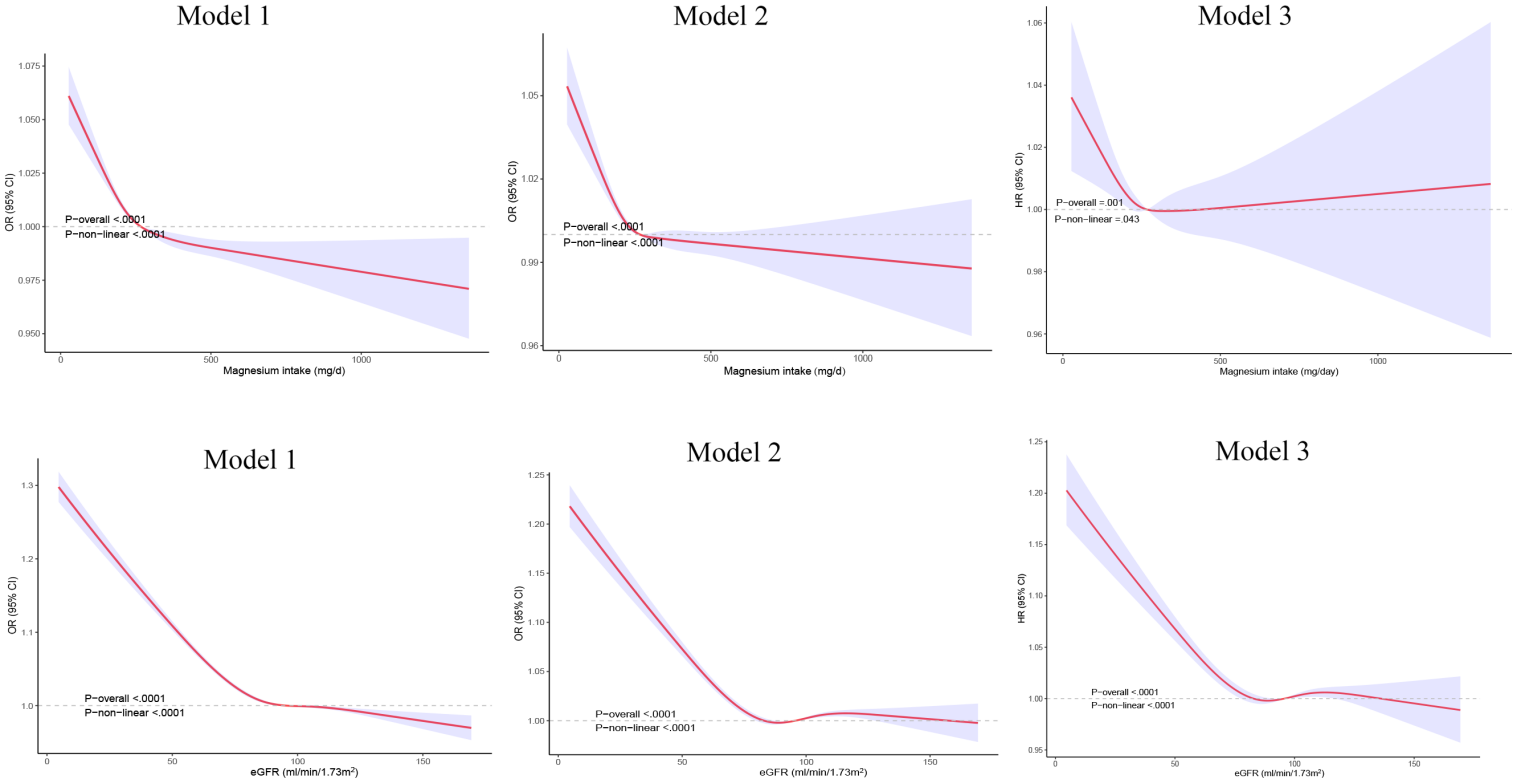
Restricted cubic spline analysis of the relationship between dietary magnesium intake, eGFR, and stroke.eGFR: estimated glomerular filtration rate. Model 1, adjusted for age and gender; Model 2, adjusted for age, gender, race, education, and the ratio of family income to poverty; Model 3, adjusted for age, gender, race, education, the ratio of family income to poverty, BMI, alcohol consumption status, smoking status, hypertension, diabetes, triglyceride level, total cholesterol level, energy intake, and dietary fiber intake, and cardiovascular diseas.

The relationship between dietary magnesium intake, eGFR, and stroke was also explored through non-linear association analyses. Using restricted cubic splines, we found that the favorable impact of magnesium on reducing stroke risk plateaued when the dietary intake exceeded ~300 mg/day after adjusting for all covariates (*p*-nonlinearity = 0.043, Figure 2). Additionally, the multivariable-adjusted spline regression model confirmed a non-linear negative correlation between eGFR and stroke risk (*p*-nonlinearity <0.0001, Figure 2).

### 3.4 Gender-stratified subgroup analysis of the association between dietary magnesium intake and stroke

Given the differences between the National Academy of Medicine’s recommendations for dietary magnesium intake in males and females and our study’s cutoff point, we adjusted for gender disparities. Analyzing dietary magnesium intake by gender (Supplementary Table S1), we found females generally had lower intake than males, with 28% consuming below 254 mg/day compared to 17% of males. Subsequently, we conducted a gender-stratified sensitivity analysis, revealing a consistent relationship between dietary magnesium intake and stroke risk, particularly among females with intake levels below 254 mg/day (Supplementary Table S2). Finally, categorizing participants based on the recommended intake of 310 mg/day, we presented gender-specific distributions and sensitivity analysis results in Supplementary Tables S1, S2, consistent with findings using the 254 mg/day cutoff.

## 4 Discussion

In this study, we identified a potential interaction between inadequate dietary magnesium intake and low eGFR, suggesting a synergistic effect on stroke risk. Further analysis revealed a significant interaction between stage G3 CKD and low dietary magnesium intake in influencing stroke risk, while similar results were not observed in stages G4 and G5 CKD, which could be attributed to the limited number of participants with stages G4 and G5 CKD in our study population. In the subgroup analyses based on sex, age, and race, the interaction between low dietary magnesium intake and low eGFR consistently emerged as a significant risk factor for stroke in participants aged ≥60 years, females, non-Hispanic Black individuals, and people of other races. Finally, dose–response and extent-response analyses indicated a non-linear inverse association between dietary magnesium intake and eGFR with stroke risk.

This is the first large-scale study to evaluate the relationship between dietary magnesium intake and eGFR, and their combined effects on stroke risk. Earlier surveys showed that 64–67% of adults in the US consumed less than the recommended amount of magnesium from dietary sources. Although magnesium intake increased in the subsequent years, it was still below the recommended levels in 53–56% of the adults between 2005 and 2006 (35). Previous studies have reported a correlation between higher dietary magnesium intake and a reduced risk of cardiovascular diseases, such as coronary heart disease and hypertension (12, 36, 37). Moreover, consistent negative associations have been observed between total dietary intake and total stroke incidence, as well as between magnesium intake and total stroke incidence (38). Consistent with these studies, we found that individuals with low dietary magnesium intake had a 1.17-fold higher stroke risk than those with normal intake (16), and the risk of stroke declined with increased magnesium intake. Although the exact mechanism underlying this correlation remains unclear, multiple pathophysiological pathways may be involved. The cardioprotective effects of magnesium may stem from its antihypertensive properties, which influence blood pressure regulation, vascular tone, and reactivity (39). Magnesium deficiency might accelerate the development of atherosclerosis through inflammation and oxidative stress, eventually leading to stroke (40, 41). In patients with stable coronary artery disease, reduced intracellular magnesium may be associated with increased thrombosis, and magnesium may potentially mitigate stroke risk by regulating blood pressure (42).

Previous studies have reported that eGFR less than 60 mL/min/1.73 m^2^ correlates with an increased risk of stroke (21). In addition, several reports, including two systematic reviews, have confirmed a linear relationship between eGFR and stroke incidence (43, 44). Nevertheless, treating eGFR as a dichotomous or continuous variable often overlooks potential non-linear relationships and regression dilution bias. In this study, we used various approaches to analyze eGFR, including restricted cubic spline analysis for its continuous form and multiple logistic regression analysis for its dichotomous classification. Our results consistently demonstrated a strong correlation between eGFR and the increased risk of stroke. The findings not only support a non-linear correlation between eGFR and stroke risk but also underscore a robust non-linear relationship that was validated through multiple methods. Abnormal renal function may increase the likelihood of stroke by influencing brain regulation, inducing cerebrovascular remodeling, and reducing cerebral blood flow (45). In addition, a decrease in eGFR is indicative of disorders like micro-inflammation, endothelial dysfunction, oxidative stress, and increased aortic pressure, which can increase the risk of atherosclerosis and cerebrovascular diseases (46).

Subsequently, we investigated the interaction between dietary magnesium intake and eGFR in the context of stroke risk. Further exploration was conducted based on the proportion of patients in each of the different CKD stages to examine the interaction between different CKD stages and dietary magnesium intake in stroke risk. The results revealed that individuals with decreased dietary magnesium intake and declining eGFR faced an increased risk of stroke, highlighting a synergistic effect between these two factors. Further analysis indicated a significant interaction between low dietary magnesium intake and stroke risk among patients with stage G3 CKD. Additionally, subgroup analyses, which included specific demographics, such as non-Hispanic Black individuals, individuals aged ≥60 years, and females, confirmed the significance of the interaction between low dietary magnesium intake and low eGFR in elevating stroke risk. It is worth noting that the interaction between low magnesium intake and low eGFR in stroke risk was observed only in females, possibly due to the generally lower dietary magnesium intake in females. In the US, females generally consume less magnesium than males and have a higher likelihood of experiencing stroke during their lifetime (47, 48). Therefore, females appear to be more responsive to the benefits of magnesium in reducing stroke risk. The association was inconclusive in other age groups, most likely due to the limited number of stroke cases in the younger population. A retrospective cohort study of 311 patients with non-dialysis-dependent CKD revealed an increased risk of end-stage renal disease (ESRD) in those with high phosphate and low magnesium levels compared with those with high phosphate and high magnesium levels (26). Furthermore, hypomagnesemia was associated with post-kidney transplant complications, such as diabetes mellitus and graft loss, supporting the renal protective effect of magnesium (26). Renal protection may help reduce the risk of cardiovascular and cerebrovascular diseases, indirectly supporting our observation of an interaction between low dietary magnesium intake and eGFR in increasing stroke risk. Several mechanisms potentially support the interaction of magnesium deficiency and low eGFR in the context of vascular complications. For instance, magnesium protects renal function by inhibiting vascular calcification through the Wnt/β-catenin pathway, and it is known to prevent kidney damage caused by hyperphosphatemia (49). In addition, given that the reabsorption of magnesium in the kidneys plays a key role in its homeostasis, a reduced GFR may lead to magnesium deficiency (50). Experimental evidence suggests that increased dietary magnesium intake and supplementation can improve renal function, reverse vascular calcification, and lower serum phosphate and parathyroid hormone levels (25, 51). Thus, magnesium deficiency may impair kidney function and reduce magnesium absorption from the urine, consequently heightening stroke risk.

In our study, most CKD patients were classified as stage G3, with fewer patients in stages G4 and G5. The analysis revealed a significant interaction between low dietary magnesium intake and stroke risk only in patients with stage G3 CKD; similar findings were not observed in patients with stage G4 and G5 CKD. This may be attributed to the smaller sample sizes of patients with G4 (270 patients) and G5 (114 patients) CKD. Additionally, special attention to magnesium intake is warranted in CKD patients, especially those in advanced stages and undergoing dialysis, as impaired kidney function may lead to compromised magnesium excretion. Excessive magnesium supplementation may result in hypermagnesemia, necessitating cautious use of magnesium supplements. Hence, it is paramount to stress that the utilization of dietary magnesium supplements should be guided by medical professionals and registered dietitians. Future research should explore the impact of magnesium intake on stroke risk in patients with CKD of different stages, as well as the safety and efficacy of magnesium supplementation.

This is the first NHANES-based study to explore the interplay of dietary magnesium intake and eGFR in stroke development to mitigate stroke risk in patients with CKD. However, this study has some limitations, including potential confounding factors, questionnaire bias, and the cross-sectional design. In this study, eGFR values were used to diagnose CKD. Future research should explore broader CKD diagnostic criteria for better patient identification and management. Therefore, the results should be extrapolated with caution, and further studies should be conducted to confirm the causal associations between dietary magnesium intake, eGFR, and stroke risk.

## 5 Conclusion

Low dietary magnesium intake and low eGFR may synergistically increase stroke risk, particularly when magnesium intake is insufficient in patients with stage G3 CKD. This heightened risk was consistently observed in subgroup analyses of non-Hispanic Black individuals, individuals aged ≥60 years, and females. Increasing dietary magnesium intake may potentially reduce stroke risk in patients with CKD, but further prospective clinical studies are needed to validate our findings and explore the underlying mechanisms of this phenomenon. Finally, we stress the importance of consulting medical professionals and registered dietitians before using dietary supplements, especially for individuals with CKD. Their guidance is crucial for ensuring safe and effective supplement use.

## Data availability statement

The original contributions presented in the study are included in the article/Supplementary material, further inquiries can be directed to the corresponding authors.

## Ethics statement

The studies were conducted in accordance with the local legislation and institutional requirements. Written informed consent for participation was not required from the participants or the participants’ legal guardians/next of kin because this study utilized anonymized data from the National Health and Nutrition Examination Survey, adhering to ethical guidelines following the Declaration of Helsinki. Approval was obtained from the National Center for Health Statistics Ethics Review Board, and participants provided written informed consent (https://www.cdc.gov/nchs/nhanes/).

## Author contributions

CL: Data curation, Formal analysis, Methodology, Visualization, Writing – original draft. LQ: Data curation, Formal analysis, Methodology, Software, Visualization, Writing – original draft. YZ: Data curation, Investigation, Visualization, Writing – original draft. LC: Conceptualization, Data curation, Methodology, Software, Writing – original draft. HW: Writing – original draft, Visualization, Supervision, Data curation, Conceptualization. HL: Data curation, Formal analysis, Investigation, Methodology, Writing – original draft. YT: Data curation, Investigation, Supervision, Writing – review & editing. HY: Conceptualization, Project administration, Supervision, Writing – review & editing.
