# Corrigendum: The interaction between estimated glomerular filtration rate and dietary magnesium intake and its effect on stroke prevalence: a cross-sectional study spanning 2003-2018

**DOI:** 10.3389/fnut.2025.1603936

**Published:** 2025-05-12

**Authors:** Chunhua Liu, Linan Qiu, Yuanyuan Zhang, Liping Chen, Huaqiang Wang, Huajian Lin, Yongjun Tao, Haiqin Ye

**Affiliations:** ^1^Lishui Hospital of Traditional Chinese Medicine Affiliated to Zhejiang University of Chinese Medicine, Lishui City, China; ^2^Department of Neurology, The First Affiliated Hospital of Wenzhou Medical University, Wenzhou, China; ^3^Lishui Central Hospital, Lishui City, China

**Keywords:** stroke, estimated glomerular filtration rate, chronic kidney disease, dietary magnesium, interaction

In the published article, there was an error regarding the affiliations. The author, Haiqin Ye, was affiliated to the incorrect affiliation: “Department of Neurology, The First Affiliated Hospital of Wenzhou Medical University, Wenzhou, China”. Their correct affiliation is “Lishui Central Hospital, Lishui City, China”.

The authors apologize for this error and state that this does not change the scientific conclusions of the article in any way. The original article has been updated.

