# Supplementary material for: The interaction between estimated glomerular filtration rate and dietary magnesium intake and its effect on stroke prevalence: a cross-sectional study spanning 2003–2018
Source: Front Nutr. 2024 Jun 11;11:1395685. doi: 10.3389/fnut.2024.1395685 (PMC11196627; doi:10.3389/fnut.2024.1395685)
Supplement: Supplementary file 1 [file Table_1.docx]

**Table S1.** The distribution of dietary magnesium intake is based on gender.

| Characteristic | N^1^ | Overall, N = 37,637 (100%)^2^ | Group | | P Value^3^ |
| --- | --- | --- | --- | --- | --- |
|  |  |  | Female, N = 19,329 (52%)^2^ | Male, N = 18,308 (48%)^2^ |  |
| Magnesium intake group (254 mg/day as the cutoff point), n (%) | 37,637 |  |  |  | <0.001 |
| Mg > 254 mg/day |  | 20,634 (55%) | 8,647 (23%) | 11,987 (32%) |  |
| Mg ≤ 254 mg/day |  | 17,003 (45%) | 10,682 (28%) | 6,321 (17%) |  |
| Magnesium intake group (310 mg/day as the cutoff point), n (%) | 37,637 |  |  |  | <0.001 |
| Mg > 310 mg/day |  | 13,421 (37%) | 4,945 (13%) | 8,476 (24%) |  |
| Mg ≤ 310 mg/day |  | 24,216 (63%) | 14,384 (37%) | 9,832 (26%) |  |

1 N not Missing (unweighted)

2 Median (IQR) for continuous; n (%) for categorical

3 Wilcoxon rank-sum test for complex survey samples; chi-squared test with Rao & Scott's second-order correction
