# Supplementary material for: The interaction between estimated glomerular filtration rate and dietary magnesium intake and its effect on stroke prevalence: a cross-sectional study spanning 2003–2018
Source: Front Nutr. 2024 Jun 11;11:1395685. doi: 10.3389/fnut.2024.1395685 (PMC11196627; doi:10.3389/fnut.2024.1395685)
Supplement: Supplementary file 2 [file Table_2.docx]

**Tabel S2**. Gender-stratified subgroup analysis of the association between dietary magnesium intake and stroke.

| Variable | | Model 1 OR (95% CI) | p-value | Model 2 OR (95% CI) | p-value | Model 3 OR (95% CI) | p-value |
| --- | --- | --- | --- | --- | --- | --- | --- |
| The mean intake  level served as  the cutoff point | Female |  |  |  |  |  |  |
|  | Mg > 254 mg/day |  |  |  |  |  |  |
|  | Mg ≤ 254 mg/day | 1.02(1.01-1.02) | <0.001 | 1.01(1.01-1.02) | <0.001 | 1.01(1.00-1.02) | 0.016 |
|  | Male |  |  |  |  |  |  |
|  | Mg > 254 mg/day |  |  |  |  |  |  |
|  | Mg ≤ 254 mg/day | 1.02(1.01-1.02) | <0.001 | 1.01(1.00 -1.02) | 0.005 | 1.00(0.99-1.01) | 0.4 |
| The recommended  intake level served  as the cutoff point | Female |  |  |  |  |  |  |
|  | Mg > 310 mg/day |  |  |  |  |  |  |
|  | Mg ≤ 310 mg/day | 1.02(1.01-1.02) | <0.001 | 1.01(1.00 - 1.02) | <0.001 | 1.01(1.00-1.02) | 0.046 |
|  | Male |  |  |  |  |  |  |
|  | Mg > 310 mg/day |  |  |  |  |  |  |
|  | Mg ≤ 310 mg/day | 1.01(1.01-1.02) | <0.001 | 1.01(1.00-1.01) | 0.001 | 1.00(1.00-1.01) | 0.3 |

OR = Odds Ratio, CI = Confidence Interval, eGFR = estimated glomerular filtration rate; Model 1, adjusted for age and gender; Model 2, adjusted for age, gender, race, education, and the ratio of family income to poverty; Model 3, adjusted for age, gender, race, education, the ratio of family income to poverty, BMI, alcohol consumption status, smoking status, hypertension, diabetes, triglyceride level, total cholesterol level, energy intake, dietary fiber intake and cardiovascular disease.
